# Progress in Abdominal Surgery

**Published:** 1901-07-27

**Authors:** 


### Progress in Abdominal Surgery.

**Tuberculous Peritonitis.**—Wallis[1] classifies the various theories as to the reason of the beneficial effect of laparotomy in this disease as follows :—

(1) The removal of the ascitic fluid causes an alteration in the abdominal pressure, and an increased blood supply.

(2) Exposure to air.

(3) Action of light.

(4) Introduction of toxalbumin, which is fatal to the tubercle bacillus.

(5) Traumatism of operation causes fibrinous peritonitis, and the bacilli become encapsuled.

(6) It is due to the advent of leucocytes and the resulting phagocytosis.

(7) The opening of the abdomen causes a physiological change, which in some way is detrimental to further growth of the bacilli.

In many cases of tuberculous peritonitis where the mesentery is involved, intestinal occlusion occurs, with or without the presence of a tumour. According to the case the treatment then consists in (1) the formation of a fæcal fistula; (2) resection of the bowel; or (3) lateral anastomosis or short circuit. Four varieties of abdominal tumours which are caused by tuberculous inflammation have been described:—

1. Nodular tumours of the mesentery.
2. Tumours of the omentum.
3. Thickened, contracted, and adherent coils of intestine.
4. Encysted exudation forming a tumour, with walls composed of adherent intestines, and other abdominal or pelvic viscera which happen to be adjacent to the focus of inflammation.

The third variety is the one which is apt to be most misleading, both before and after the abdomen is opened.

**Diaphragmatic Abscess.**—Godlee[2] says that the dulness at the lower part of the chest caused by this affection ought to be distinguishable from the dulness of a pleural effusion by the following signs:—1. The movements of the chest are not impaired on the affected side. 2. The upper limit of dulness is not so sharply defined. 3. Breath sounds may be heard below the level of the dulness, and, if a deep inspiration be made, the line at which the breath sounds and vocal resonance are heard and at which vocal fremitus is felt is distinctly lowered. 4. If the patient is emaciated and is examined in a good light, if the pleura is healthy the lower margin of the lung can be seen (on the right side) moving up and down during respiration. This would show that the cause of the dulness was below and not above the diaphragm. 5. Instead of dulness there may be a great extent of tympanitic resonance, and over the resonant area the bell sound may be elicited in a most perfect manner. This only occurs in cases where gas as well as pus is present, and from the similarity of the signs to those of pneumo thorax has given rise to the name of subdiaphragmatic pneumothorax. It may reach as high as the second rib or down into the iliac fossa. This condition is to be diagnosed in most cases from the genuine pneumothorax, by the fact that the movements of the thorax are not impaired.

**Rupture of the Diaphragm,** says Thomson,[3] is a very rare injury apart from stabs and gunshot wounds, which injure the diaphragm after penetrating the lateral thoracic walls. It is nearly always fatal in twenty-four or forty-eight hours, usually from simultaneous injury of internal organs causing hæmorrhage or peritonitis (stomach or intestines). The special symptoms, in addition to shock, etc., are, pain referred to the region of the stomach, superficial and rapid breathing, and the physical signs of displacement of the viscera. It is not always diagnosed during life, because the condition of the patient may not permit of the necessary examination. Interest attaches to the method in which the subcutaneous rupture is produced, and three cases are cited which support the view that the rupture is the result of a sudden and great increase in the intra-abdominal pressure, the vault of the diaphragm—as the site of least resistance—giving way like an over-distended rubber bag.

**Gunshot Wounds** of the abdomen are discussed by Martin,[4] who maintains that operations should be performed as soon as possible. The earlier the better, for each hour of delay counts against the patient. The operation should not be delayed because the patient is suffering from shock. The great mortality after gunshot wounds of the abdomen is due to septic peritonitis and hæmorrhage, and the effect of these two conditions in producing and continuing the shock; so the sooner operation is undertaken, the sooner the cause for shock will be removed, and, therefore, the greater will be the chance of saving the patient. The author speaks highly of the immense advantage of the transfusion of salt solution in these cases.

**Post-operative Intestinal Obstruction** has been treated successfully by Cleveland[5] by the insufflation of oxygen per rectum. Its use is not attended with danger if ordinary care is observed. It should be introduced slowly and as soon as the patient complains of decided pain and feeling of increased distension the gas should be cut off. It not merely has the power to straighten out the intestines and open the lumen for the passage of gas and fæcal contents, but serves as a stimulus to peristalsis. It should be used as soon as any symptoms of obstruction begin to manifest themselves.

[1] Clin. Jour., Feb. 13., 1901, p. 269. [2] Brit. Med. Jour., Oct. 6, 1900, p. 997. [3] Edin. Med. Jour., Feb. 1901, p. 173. [4] Annals of Surgery, Mar. 1901, p. 316. [5] Med. Rec., Jan 5, 1901, p. 3.

